# Resumption of professional football league with spectators during the COVID-19 pandemic: The implementation of Bio-secure bubble protocol

**DOI:** 10.5339/qmj.2022.31

**Published:** 2022-07-26

**Authors:** Abdul Wahab Al Musleh, Naushad Ahmad Khan, Sameer Abdurahiman, Mohammad Asim, Ayman El-Menyar, Gordon Penney, Hassan Al-Thani

**Affiliations:** ^1^Clinical Information Systems (CIS); Hamad Medical Corporation & Medical Affairs; Supreme Committee for Delivery & Legacy, Doha, Qatar E-mail: aymanco65@yahoo.com; ^2^Department of Surgery, Trauma & Vascular Clinical Research, Hamad General hospital, Doha, Qatar; ^3^Department of Clinical Medicine, Weill Cornell medical college, Doha, Qatar; ^4^Health Safety Security Environment; Supreme Committee for Delivery & Legacy, Doha, Qatar; ^5^Department of Surgery, Trauma and vascular surgery, Hamad General hospital, Doha, Qatar

**Keywords:** Professional football league, Bio-secure bubble, Severe acute respiratory syndrome coronavirus 2 (SARS-CoV-2), COVID-19, pandemic, Asian Football Confederation, spectators, reverse transcription-polymerase chain reaction (RT-PCR), Qatar

## Abstract

Background: Elite professional sports events involving mass gatherings carry a high risk of viral transmission during the coronavirus disease of 2019 (COVID-19) pandemic. We describe the potential impact of resuming professional football leagues involving international participants adhering to a strict *Bio-secure bubble* protocol and investigate the consequences of spectators/fan attendance at such mass events during the ongoing COVID-19 pandemic in Qatar.

Methods: We conducted a descriptive cohort study involving football players, referees, match officials, local organizing committee (LOC) members, hotel and security staff working in close coordination, and over 10,000 spectators from the Asian Football Confederation (AFC) Champions League (East) and the final match. The study covered almost four weeks of the event (November 19 to December 19, 2020) under a robust *Bio-secure bubble* protocol. It included extensive severe acute respiratory syndrome coronavirus-2 (SARS-CoV-2) RT-PCR (reverse transcription-polymerase chain reaction) every 3–6 days and clinical symptom monitoring on and off the field. Target variables included positive RT-PCR results and clinical symptom monitoring among participants, and rapid antigen testing for fan attendance to examine their safe return to the stadiums.

Results: A total of 12,250 RT-PCR tests involving 3158 individuals in the *Bio-secure bubble* were done over one month for all the AFC (East) matches, including the final match. Overall, 44 matches involving 16 teams were played. During the championship, only five individuals (three LOC members and two match officials) returned positive for COVID-19 infections. Four individuals (three team staff/officials and one person outside the Bio-secure bubble) had reactive results. None of the players tested positive for COVID-19 infection. All individuals testing positive were asymptomatic or had mild symptoms, with no one requiring hospitalization other than symptomatic treatment. The overall positivity rate was 0.15% for the entire duration of the AFC (East) Champions League. For the final match, a total of 10,320 rapid antigen tests were done for spectators, of which only one test was positive for COVID-19.

Conclusions: This report shows a very low incidence rate of COVID-19 infections during mass gathering events at the international level. For the resumption of football with spectators, careful mitigation strategies should be considered to reduce the risk of transmission to a sufficiently safe level. This may require proper coordination and measures (i.e., physical distancing, testing, entry, and exit routes in the stadium, and seating arrangement inside the stadium with limited attendance). Based on this, during the ongoing COVID-19 pandemic, the supervised and controlled resumption of football matches with spectators can be done safely provided that a strict *Bio-secure* *bubble* protocol has been implemented.

## Introduction

In March 2020, the World Health Organization (WHO) declared the coronavirus disease of 2019 (COVID-19) a pandemic.^1^ COVID-19 is an infection caused by the severe acute respiratory syndrome coronavirus-2 (SARS-CoV-2) virus with various symptoms ranging from mild to severe illness. Following the COVID-19 outbreak, by April 2020, the health authorities and governments of several countries declared confinement measures to decelerate the disease propagation, which resulted in the suspension of all professional sports training and elite competitions.^2,3^.

There has been increasing debate about the desirability of professional football resumption during the COVID-19 pandemic. These professional football tournaments involving international participants provide mass spectacles for the public^4^ while producing having significant health and socioeconomic effect on the host nation(s)^5^, including an increased risk of infectious disease transmission.^2,6^ Therefore, pandemics like COVID-19 have brought an added earnestness to assess the impact of hosting large sporting events. Due to the global outbreak of the COVID-19 pandemic, Qatar's professional football leagues were also interrupted after March 2020. With each passing day, more information about viral transmission mechanisms is emerging and becoming more apparent. Preventive measures have become the mainstay of addressing individual-level risk control. However, these measures sometimes become impractical and difficult to implement when dealing with sports involving mass gatherings and elite athletes, making physical distancing challenging to follow^7,8^. It also involves international travel and spectators, further potentiating the risk of viral spread.^9,10^

Over almost one and a half years, the global football community has worked diligently to find solutions for the sport's safe return amidst the global COVID-19 pandemic, and in the second half of 2021, it appears that the great sports freeze has thawed.^11,12^ Qatar, a country in the Arabian Gulf with a total population of 2.8 million, has also played an instrumental role in building confidence among players and spectators alike. It safely hosted a couple of football tournaments with players and spectators in attendance with infection control measures.^13–15^ In May 2020, the Qatar government created a task force composed of sports physicians, scientists, and healthcare professionals under one umbrella to allow the resumption of the football league and implement a return-to-competition protocol. It was a massive challenge considering that the WHO suggested that hosting an event involving a mass gathering during the active phase of the ongoing pandemic be of very high risk.^10,12^

**Figure 1.**
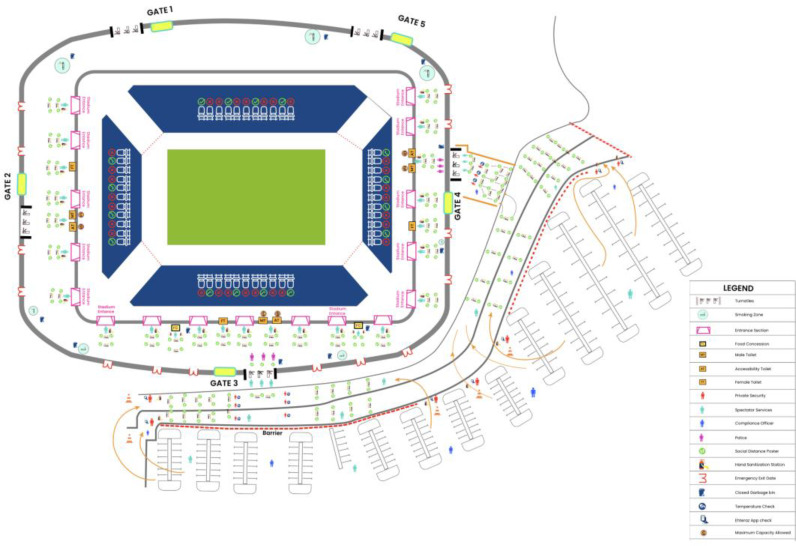
Seating scheme for spectators with implementation of organizational precautions to minimize the risk of transmission of COVID-19 infections inside the stadium during the championship

Over the six months (November 2020–April 2021), Qatar has hosted the Asian Football Confederation (AFC) Champions League West and East Zone matches, including the final, the Amir cup in 2020. In February 2021, it concluded with hosting the FIFA (Fédération Internationale de Football Association) Club World Cup (FCWC), with spectators permitted to attend and stadiums operating at 30 percent capacity. Qatar has become one of the first countries to implement an expanded *Bio-secure bubble* system by including many team competitors for several sports events with spectators during 2020 and 2021. The successful and safe return of the continental football league in Asia was marked by the initial west group stage matches of the AFC competition, played without spectators, under strict adherence to *Bio-secure bubble protocol*. A Bio-secure bubble is a sanitized area that operates in a strictly controlled environment involving the interaction between a specific set of people, all of whom need to test negative for COVID-19 by the reverse transcriptase-polymerase chain reaction (RT-PCR) test. The isolated space remains closed to outsiders. Nobody can leave or enter once the bubble is in effect. The Bio-secure bubble system included regular COVID-19 testing, safe transportation methods, and regular disinfection of tournament venues, including training and media facilities for the safe and efficient return of the first professional football league involving spectators. Under this protocol, Qatar hosted 60 West Zone matches in the 2020 AFC Champions League successfully and safely. It boosted the organizers’ confidence to host 44 AFC Champions League (East) matches in November and December 2020, apart from the final, which saw more than 10,000 spectators’ safe attendance. The event welcomed and marked the safe return of spectators to the sporting arena. Requirements expected of the spectators included double vaccination (second vaccine at least 14 days before), a recent negative PCR test, recovery from COVID-19 infection (14 days before), physical distancing (seats with two empty seats in between them; Figure 1) and always wearing a face mask.

There is a gap in the existing literature about the impact of mass outdoor events like football matches involving intercontinental participation and patterns associated with spectator attendance on the possible spread of COVID-19 infections. From a public perspective, the resumption of football and other mass gathering events along with spectators by implementing multifaceted infection risk mitigation approaches is imperative, as football represents such a significant mass outdoor hobby across many Middle Eastern countries and worldwide. This study describes the success of the return-to-competition protocol, involving the clinical and safety measures implemented in the *Bio-secure* system. We also report the consequences of the operational plans undertaken for the resumption of the international football league with spectators in stadiums for the first time in Qatar during the active phase of the COVID-19 pandemic.

## Methods

This is an observational cohort study including players, staff, and referees of the AFC Champions League 2020 (East Zone), Qatar Football Association (QFA), along with local organizing committee (LOC) staff, hotel staff, state security personnel outside the Bio-secure bubble system and the spectators. The AFC Champions League (East) and its final match were held from November 18 to December 19, 2020, in Qatar. Each team typically consisted of 45–60 persons, including players, staff, and family members. The AFC (East) Champions League included 16 teams over 30 days, with the first match on November 18 and the last game played on December 13, 2020, and the final match played between the champions of the AFC (West) and the AFC (East) on December 19, 2020. This article follows the Strengthening the Reporting of Observational studies in Epidemiology (STROBE) checklist (**Supplementary Table**).

### “Bio-secure Bubble Protocol” for the players, match officials, and staff during the Championship League

In May 2020, the Qatar Sports League, in conjunction with the Ministry of Public Health (MoPH), created a task force composed of sports physicians, scientists, and government officials to develop guidelines to resume football activities. This task force established a framework based on scientific evidence to reduce the health risks of returning to international professional football amidst the ongoing COVID-19 pandemic. A robust and strict protocol was implemented wherein players, and officials were part of a strict “*Bio-secure bubble Protocol*”^7,16,17^ Intercontinental sports require international players’ participation, which involves frequent travel, and bio-bubbles create a safe environment. The mainstays of this *Bio-secure bubble* system were strict adherence to the protocol and regular testing.

Upon arriving at Hamad International Airport, all international players and tournament officials were tested for COVID-19 infections. Participants completed their immigration process through dedicated counters and were transported to their accommodations. Participants were quarantined in their hotel rooms until their test results were confirmed as negative to participate in the event. After they arrived at their designated accommodation/hotels, players and tournament officials were placed in a “*Bio-secure bubble*.” This limited their movement only to and from their designated accommodations, stadiums, and training sites. Each team was assigned a dedicated meeting room with a seating arrangement plan to ensure physical distancing for team meetings. Food and refreshments were served to each player/official/staff in their rooms. Every team had a pre-scheduled time for gym sessions for physical training-related activities. All pieces of equipment were sterilized before and after use. Individuals under the same bubble were not permitted to contact anyone outside their bubble.

The bubble protocol mandated that all teams be transported in small groups at a maximum of 50 percent vehicle capacity using alternate seats and maintaining the 1.5 meters separation distance. All stadiums were equipped with medical clinics and a medical staff team to ensure a swift and well resourced, frontline response during the tournament. All medical staff was tested for SARS-CoV-2 virus by RT-PCR, 24 hrs. before the match and were not a part of the Bio-secure-bubble protocol. Stadiums also had a dedicated isolation facility to hold individuals exhibiting COVID-19 symptoms or requiring medical assistance. Players and team officials underwent regular temperature checks throughout the tournament before entry into tournament-related venues. All players, support staff, and officials were required to adhere to these measures; violations were subject to disqualification from the league.

### COVID-19 testing protocol for players and match officials during the Championship League

The current gold standard for detecting the presence of COVID-19 infection is the PCR test.^18,19^ The test is highly sensitive and specific to SARS-CoV-2 viral RNA.^20^ All international participants (including officials) were required to have a negative PCR test for COVID-19 infection within 48–72 hours before entering the country. For national participants, testing was performed 1–2 days before entering the bubble hotel or 1–2 days before the competition. Subsequent swabbing for COVID-19 PCR testing was undertaken from all participants every 3–6 days until the individual participated in the event.

### Return-to-stadium protocol for spectators

The AFC (East) Champions League marked the partial return of spectators. Ticket allocations were limited to 30% of the overall capacity of each stadium. Crowd management poses the most significant challenge during sporting events. The AFC (East) Champions League made special arrangements to mitigate COVID-19 transmission during the event by implementing physical distancing measures, such as reducing the overall number of attendees, using mobile ticket sales, and staggering and controlling the arrivals and departures. All spectators had to undergo rapid antigen tests up to 72 hours before each match at approved facilities designated by the MoPH. Only spectators with negative SARS-CoV-2 antigen tests were allowed to attend the match. Before entering the stadium, an initial health screening such as a temperature check was conducted on each spectator. All spectators had to wear a facemask throughout the event. Seating inside the stadiums was staggered to provide adequate physical distancing, and spectators were spaced apart by at least 1.5 meters in all directions to avoid crowding (Figure 1). Family members of the same household could sit together. Nobody should leave their seating row during the match to minimize interactions. Spectators were asked to refrain from shouting and behaving boisterously to celebrate during the game to mitigate any chance of droplet transmission. Before each event, all high-touch surfaces inside the stadiums were disinfected daily, and multiple mobile hand-wash stations and automated hand sanitizer dispensers were installed to the designated areas in the stadiums. The spectator restrooms were adequately disinfected, and sanitary staff ensured high-touch surfaces in the washroom, including doors, faucets, and paper towel dispensers, were disinfected at frequent intervals. These venue-specific practices were repeated before all matches. A comprehensive multilayered protocol was strictly implemented while resuming international soccer events with spectators.

### PCR analyses and interpretation protocol

Following standardized protocols, all PCR analyses were performed at the Communicable Disease Centre Laboratory. Nasopharyngeal or oropharyngeal swabs (Huachenyang Technology, China) were collected. All assays were pre-validated before routine use. PCR testing was performed on aliquots of Universal Transport Medium. Aliquots were extracted on the Qiagen (QIA) symphony platform (QIAGEN, USA) and tested with real-time reverse transcription PCR (RT-qPCR) using the TaqPath COVID-19 Combo Kit (Thermo Fisher Scientific, USA) on an ABI 7500 FAST (Thermo Fisher, USA). Then, a custom protocol^21^ was used on a Hamilton Microlab STAR (Hamilton, USA), and aliquots were tested with the AccuPower SARS-CoV-2 Real-Time RT-PCR Kit (Bioneer, Korea) on an ABI 7500 FAST, or loaded directly into a Roche Cobas® 6800 system. Next, they were assayed with the Cobas® SARS-CoV-2 Test (Roche, Switzerland). The results were interpreted as per the manufacturer's instructions based on the respective cycle threshold (cT) of the gene target amplified. Results were reported based on the cT value either positive (cT < 30), reactive (40 > cT > 30), negative, or inconclusive. Reactive samples were defined as cases where the possibility of transmitting the infection was relatively low. The average time required from when the sample was taken for PCR testing to the validation and availability of results was approximately six hours.

### Rapid Antigen Test analyses and interpretation for spectators

**Figure 2.**
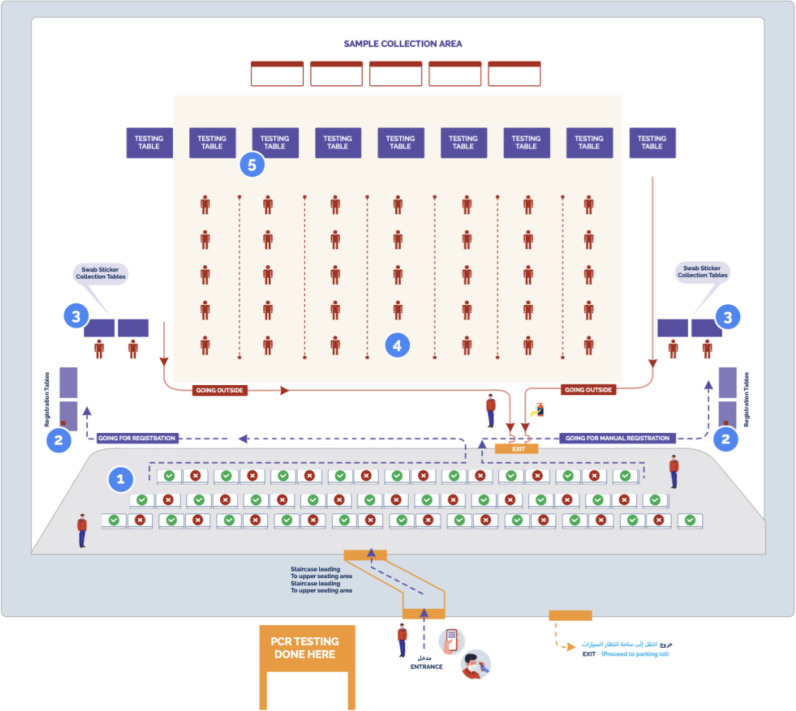
Rapid antigen testing plan for spectators

The SARS-CoV-2 rapid antigen test is a reliable, chromatographic immunoassay for the qualitative detection of specific antigens of SARS-CoV-2 in the human nasopharynx.^22^ The test provides specificity of 99.2% and a sensitivity of 95.5% (cT value ≤  30) with a testing time of 15–30 minutes. Test results were analyzed and interpreted as per the manufacturer's instructions. In the current study, the sampling was conducted to diagnose COVID-19 through rapid antigen testing among the spectators. All antigen testing was performed at two approved facilities designated by the MoPH, i.e., the Al Shahaniya Workers Camp and Qatar National Convention Centre, maintaining proper physical distancing. All the testing arrangements for spectators were organized for them by the Supreme Committee of delivery and legacy. An outline of the Rapid Antigen Testing protocol is illustrated in Figure 2. Spectators with a negative SARS-CoV-2 antigen were not required to undergo further PCR testing. SARS-CoV-2 specific antigens were measured with a nasopharyngeal swab (SARS-CoV-2 Rapid Antigen Test Kit, Roche Diagnostics International AG, Rotkreuz, Switzerland).

### Data management, monitoring, and reporting inside the Bio-secure bubble

All COVID-19 specific PCR results for the event officials (i.e., LOC members, hotel staff, security staff, and personnel outside the Bio-secure bubble system) were made available via the national COVID-19 tracking phone application (EHTERAZ), which is mandatory for all Qatari citizens and residents as one of the measures implemented in Qatar to control the spread of the virus.^23^ Additionally, team physicians also communicated the test results to the players and team officials. If any player/team official, LOC, or hotel staff was positive for COVID-19 infection at the hotel, they were immediately isolated in their room. The compliance officer, a medical doctor, and an accommodation lead were informed about ascertaining contact tracing. Anyone tested positive for COVID-19 infection inside the stadium or en route to the stadium was immediately removed, transported to an isolation facility, and PCR tests were repeated within 48–72 hrs. The COVID-19 positive individuals were retested on day three and day nine and, if tested negative or reactive, were released from the isolation facility on day 10. However, if the results were positive, the patient continued in isolation until 14 days. Discharge certificates and negative PCR certificates were issued to the participants before their departure. In cases of inconclusive reports, players and event officials continued to the matches and training sites until the availability of the retest results. The departure protocol required all participants to take a COVID-19 test 48 h before departure. The test results and certificates were emailed individually to team doctors, the AFC committee, referees, and individual email addresses for media and guests.

## Results

### COVID-19 PCR-confirmed infections and pandemic activity in Qatar

**Figure 3.**
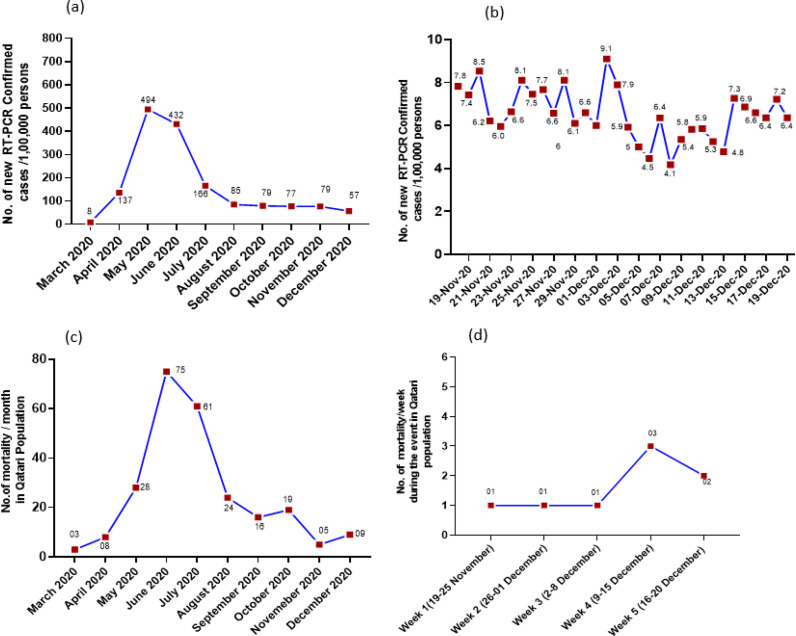
Number of new positive COVID-19 cases confirmed by RT-PCR on a monthly basis since the outbreak of pandemic till December 2020 (a), number of new RT-PCR-confirmed cases during the championship period (b), number of mortalities recorded in the Qatari population from March 2020 to December 2020 (c), and number of mortalities observed on a weekly basis during the championship league (d). The incidence was estimated per 1, 00,000 of the Qatari population (2.8 million residents) during the period of AFC (East) Champions League and Final (November 19–December 19. 2020). Data was extracted from the Qatar Open Data Portal (data.gov.qa)

By the time the AFC (East) championship and final matches were organized, the incidence of ongoing COVID-19 infections in Qatar had stabilized, with a gradual decline in the daily COVID-19 positive cases being recorded (Figure 3a). During the first day of the championship league (November 18, 2020), the total number of newly PCR-confirmed cases of COVID-19 infections in Qatar was eight per 100,000 persons (Figure 3b). This number steadily declined to six per 100,000 persons at the end of the tournament (December 19, 2020). The average incidence of COVID-19 positive infections in Qatar was 6.5 per 100,000 persons during the study period. The data is based on laboratory results of 306,129 tests, of which 5417 were positive or reactive (1.68%) when the championship was held (November 18 to December 19, 2020). The number of deaths per month among the general population in Qatar was five and nine in November and December 2020, respectively. During the championship period, the total number of deaths was recorded in the Qatar population (Figures 3c & d). Population reference data were obtained from the MoPH and the Qatar open data portal (data.gov.qa), which furnishes open access to important statistical information from government entities.

The incidence of COVID-19 infections, if any, during the AFC (East) Champions League was documented to determine the effectiveness of the Bio-secure bubble protocol on the recommencement of professional football tournaments. Besides, some players and team members regularly joined and left the team as the championship progressed. The AFC conducted 12,250 PCR tests over one month by working closely with the QFA, the medical teams, and the LOC. Altogether, 3158 individuals were tested at least once, with an average of four tests per person throughout the championship.

There were five PCR positive and three reactive cases during the championship involving match officials (n = 2) and LOC (n = 3) members. None of the players and referees tested positive for COVID-19. Local medical authorities and team doctors followed up on all positive cases. The overall positivity rate for COVID-19 among the individuals involved in the championship was 0.15% during the tournament, as shown in Table 1. Individuals who had positive COVID-19 tests were asymptomatic or had mild symptoms, and none of them required hospitalization. Six individuals, including one player, one official, and four LOCs, had experienced mild symptoms, such as body aches, fever, diarrhea, weakness, or cough. However, the PCR tests were negative for the SARS-CoV-2 virus on the same day.

### COVID-19 antigen test and infections among spectators

In the current study, to diagnose COVID-19 among the spectators, all the rapid antigen testing was performed at two approved facilities designated by the MoPH in which proper physical distancing was maintained. A total of 10,320 rapid antigen tests were done for the final match. Only one (n = 1) spectator returned a positive result for COVID-19 infection during the final event.

Notably, no spike in COVID-19 cases was detected within a week after the football match. Hence, there might not have been any significant (if any) transmission of the virus among spectators.

## Discussion

Qatar was one of the first among Middle Eastern countries to implement a robust Bio-secure bubble protocol for the resumption of intercontinental professional football with spectators. Qatar set the benchmark in hosting tournaments involving multiple international teams, across various venues, and under a strict *Bio-secure bubble* protocol, by successfully hosting the AFC Champions League West and East Zone matches up to the final itself.

This is the first study from the Middle Eastern region to report the consequences for the players, spectators, and hosting country of a controlled resumption of the international football championship league amidst the COVID-19 pandemic. Our study demonstrated that the resumption of professional football under strict adherence to the *Bio-secure bubble* protocol and a gradual return of a limited number of spectators in stadiums were not associated with COVID-19 infections under the epidemiological circumstances prevailing during November and December 2020 in Qatar. A comprehensive safety net of expert planning, vigorous testing, and medical protocols was implemented for the AFC Champions League (East) held in Qatar to ensure the health protection of all stakeholders, including players, match officials, and spectators. The extensive PCR-based surveillance during the Bio-secure bubble protocol demonstrated no evidence of club-to-club or community transmission. During the entire championship, our extensive surveillance program proved that elite intercontinental football could be safely resumed during a pandemic with strict adherence to a Bio-secure bubble protocol that mitigates the transmission risks among players, staff, and local personnel, Implementation of this protocol also had significant financial implications regarding the high costs involved for performing all Bio-secure bubble-related operational measures.

Our findings are consistent with those from the German Bundesliga professional football league. They reported a successful return of the game under a controlled environment with a diminished risk of viral transmission.^24^ The authors reported eight positive cases of COVID-19 infections among 1079 players (0.74% positivity rate) and four positive cases among 623 match officials over nine weeks (May to July 2020). Overall, they reported only 12 positive cases (0.7%) among 1702 regularly tested individuals. They also observed a population infection rate of only five per 100,000 per week among the general population during the event.^24^ In our study, none of the players returned positive test results. We found five PCR positive or reactive cases (0.15% positivity rate) among 3158 individuals tested during the championship period.

It should be noted that the football league was organized at a time when there was low community incidence rate (8 positive cases per 100,000), and the incidence of the COVID-19 had stabilized in Qatar since March 2020. Furthermore, the incidence in the community steadily declined to six per 100,000 persons at the end of the tournament (December 19, 2020). Also, it is worth mentioning that more than 99% of the participating players and other officials maintained their negative COVID-19 status during the championship league, indicating that no transmission of SARS-CoV-2 had occurred. This agrees with the observation that disease transmission during match play itself is not very high^2^, and there may be a possibility of involvement of some indirect methods. However, we must acknowledge that even in countries where the surge of COVID-19 infections has initially been contained, a subsequent wave of new infections involving new strains has been reported.^25,26^ The *Bio-secure bubble* protocol ensures more robust infection mitigation measures in such a scenario. It assumes greater importance in creating the safest possible environment for the return of professional football competitions.

A previous prospective cohort study in Qatar by Schumacher et al.^27^ had reported that 85 subjects (6.4% positivity rate) returned positive for COVID-19 among 1337 participants (football players, officials, and staff), of which 36 (2.7% positivity rate) were players. As a part of the Qatar Sports League, this event was hosted without spectators between June 8, 2020, and September 2, 2020, in Qatar. The infection rate of COVID-19 was high at that time (47 per 100, 000 persons on the first day of the event [June 8, 2020]) and was consistent with that of the general population during the same period. The high positivity rate of COVID-19 infection among players and others in this study could be because the event was being played during the very active phase of the COVID-19 pandemic when the transmission risk was relatively higher. In mid-May–July 2020, COVID-19 cases in Qatar were doubling every week, suggesting that Qatar was on a transmission trajectory similar to many European countries. In late June 2020, however, daily case numbers began to plateau in Qatar, and by early November 2020, cases were dramatically reduced. We cannot extrapolate our study's findings to other settings, as the data presented in our study corresponded to when the COVID-19 incidence rate had stabilized in Qatar. The risk of infection transmission was relatively low in the general population. Nevertheless, it is imperative to pinpoint the importance of following a close vigil on the hosting country's prevailing safe epidemiological conditions when implementing a return-to-play protocol designed to mitigate the risk of SARS-CoV-2 transmissions.

In our study, no player, staff member, or match official required hospitalization or urgent medical treatment following the diagnosis of COVID-19 infection other than symptomatic treatment. They only manifested mild symptoms (sore throat, headache, others). Our data are consistent with the findings from previous sports events conducted in Qatar.^27^ In the present study and a previous report^26^, most individuals who tested positive for COVID-19 were young, asymptomatic, experienced mild symptoms, and had no significant comorbidities.^28^

Since the outbreak of the COVID-19 pandemic in 2019, few reports of sporting events^29,30^ other than football^31^ have documented the transmission of COVID-19 cases during these events. As such, evidence is still lacking on whether COVID-19 measures and the impact of the virus on fan presence can mitigate the super-spreader nature of mass sporting events. One study showed that following a day after an indoor hockey match in Florida, USA (July 2020), a player was infected with COVID-19 infection, ultimately transmitting it to other players and staff. Fifteen of 22 players and one rink staff member tested positive or experienced symptoms.^32^ Another study revealed a cluster of five COVID-19 cases linked to playing squash at a sports venue in Maribor, Slovenia. It was assumed that the transmission occurred indirectly through contaminated objects in changing room or squash hall or via aerosolization in the squash hall.^33^ A US study found that each National Basketball Association and National Hockey League game in March 2020 contributed to 380 more COVID-19 cases and 16 more deaths per one million people in the counties where these events occurred.^2^ AFC Champions League resulted in only a few COVID-19 positive cases because matches were played in outdoor settings, where physical contact between the players was limited and brief.

### Influence of the limited attendance of Spectators on the championship league

Outdoor events, such as professional football involving mass gatherings, can act as super-spreaders of an airborne virus like COVID-19. Moreover, there are unprecedented challenges to COVID-19 mitigation strategies in stadiums due to the involvement of many factors, such as the high number of spectators, seating proximity, and the high level of contact between athletes. Ahammer et al.^2^ and Cardazzi et al.^34^ determined the impact of sporting events as super-spreaders. Ahammer et al. looked at mass indoor events during the COVID-19 pandemic and found that these events led to around 380 more COVID-19 cases and 16 more deaths per one million people. Currently, there is a lack of evidence on the impact of spectators’ attendance under a controlled environment on the mass sporting events during the COVID-19 pandemic. During the championship league's final event, only one spectator returned with a positive result for COVID-19 infection after rapid antigen testing. The case was detected before he/she could attend the match and hence never went to the stadium. A study with a similar intent looked at the impact of the virus's spread from English football matches played in February and March 2020, before the first national lockdown. The evidence suggested that regardless of how full the stadiums were, the health outcomes after an English football match in March were consistent; for every 100,000 people in the same local area, there were six COVID-19 cases, two deaths, and three overall deaths.^35^ In contrast to the above findings, our study has shown that when multilayered protection involving physical distancing, disinfection, communication, and crowd control is strictly implemented, it is possible to resume professional football with limited spectators in stadiums amidst the COVID-19 pandemic. Also, results suggest caution in returning to spectator attendance at matches. In our setting, the final match of the AFC East Championship was played with 30% attendance.

Despite studies showing that antigen testing is accurate for detecting positive COVID-19 cases, no factual inference can be drawn from the current data regarding the potential risk of SARS-CoV-2 transmission during activities involving spectators due to the possibility of false-negative results. The latter is likely to raise testing concerns. Any positive antigen result will necessitate a PCR confirmation test, but a false-negative antigen test could theoretically open the door for the overlooked spread of the infection. Other spot confirmatory tests (i.e., Lateral Rapid Flow Testing) on random negative antigen samples can be a safeguard; however, it is cost-ineffective to do both tests for almost all spectators. Daily testing should be done as an added layer of surveillance protection when we intend to include spectators in sports events.

When the COVID-19 pandemic first emerged at the end of 2019 into the start of 2020, few could have anticipated that the COVID-19 crisis would stretch into 2021. The resumption of sports involving mass gatherings amid the COVID-19 pandemic remains debatable. The pandemic continues to surge, and many experts remain concerned about forging a safe environment for the athletes and staff. Nevertheless, many international sporting events are forging ahead. Qatar has demonstrated a safe and gradual resumption of international football tournaments and other sports activities, like tennis, equestrian, golf, and the Qatar motorcycle Grand Prix, by collaborating across local and international sports bodies at a time when a professional sports activity without spectators seemed to be the norm everywhere (Table 2).

Perhaps the robustness and effectiveness of Qatar's expanded *Bio-secure bubble* sports system for the AFC Champions League tournament with and without spectators will lead to new ways of conceptualizing how sports involving mass gatherings can be organized during a pandemic.

The current manuscript was written when much about the future of sports involving spectators remains uncertain, as seen from a specific set of perspectives and circumstances arising from the COVID-19 global pandemic. At this manuscript writing, a vaccination program has already been initiated in Qatar, and 88.3% of the Qatar population aged 16 years and above had already received two doses of the vaccine, with more than 6.4 million doses administered.^36^ The vaccination program remains the biggest hope to direct a series of pilot and research projects like the one discussed in the current study (the *Bio-secure bubble* protocol). It is expected to serve as a model for sports leagues to resume with spectators. In addition, it will ensure safety and provide a scheme for the public to overcome the fear of being in large crowds after such an extended period of being physically distanced. It could act as a catalyst to win back the support of an increasingly skeptical football fan. Nevertheless, we have demonstrated that the return of spectators to the stadium is possible. However, it would depend on the transmission rates of the virus and the epidemiological milieu of the hosting country.

### Limitations

One limitation of the current study was its retrospective nature. Also, we could not follow up with the spectators to determine the post-event transmission rate. The post-event epidemiological data did not show any spike in the local transmission of COVID-19 cases (Figure 3a). Nevertheless, the efforts and conscientiousness of various stakeholders during the COVID-19 pandemic outbreak and extensive health and safety protocols enabled the safe resumption of football with thousands of supporters in the stadium despite the challenges posed by the ongoing pandemic.

## Conclusions

This report demonstrates that the resumption of professional football activities at the international level under strict adherence to the *Bio-secure bubble* protocol and the gradual return of spectators in stadiums is not associated with a substantial spread of COVID-19 infection. The safety of this protocol will be fortified further by the current vaccination programs. Qatar's recent preparation, strategies, and successful experiences for mitigating infection during professional football tournaments involving international participants were critical in restarting sporting events with or without spectators. The game's safe and infection-free hosting marks an important milestone in Qatar's preparations for the FIFA World Cup 2022. It provides an opportunity to test its operational plans further and ensure its readiness for football's showpiece event in 2022 amidst the world's uncertain epidemiological circumstances.

### Ethics approval and consent to participate

This study was granted ethical approval from the medical research center and institutional review board of Hamad medical corporation, Doha, Qatar (IRB#MRC-01-21-431).

### Consent for publication

not applicable

### Availability of data and material

All data generated or analyzed during this study are included in this article. Data are accessible upon agreement with the Medical Research Centre, Hamad Medical Corporation, Doha, Qatar.

### Competing interests

None

### Funding

None

## Figures and Tables

**Table 1:** Distribution of individuals participating in the AFC Champions League (East) based on COVID-19 RT-PCR results

	Negative Cases	Positive Cases	Reactive Cases	Inconclusive	Total (n = 3158)

Sports Persons	388 (99.7%)	0 (0%)	0 (0%)	1 (0.3%)	389

Referees	16 (100%)	0 (0%)	0(0%)	0 (0%)	16

Team Staff/Officials	500 (93.1%)	2 (0.37%)	3 (0.55%)	2 (0.37%)	537

Family Members	7 (87.5%)	0(0%)	0(0%)	1 (12.5%)	8

Local Organizing Committee (LOC)^*^ Staff	1186 (99.2%)	3 (0.25%)	0(0%)	6 (0.50%)	1195

Hotel Staff	387 (98.7%)	0 (0%)	0 (0%)	5 (1.3%)	392

State Security Personnel	98 (98.9%)	0 (0%)	0 (0%)	1 (1.01%)	99

Personnel outside the Bio-secure Bubble System	521 (99.8%)	0 (0%)	1 (0.19%)	2 (38.3%)	522


Positive cases were detected by reverse transcriptase-polymerase chain reaction (RT-PCR) based on cT value*. Positive for COVID-19 virus; cT value <  30; Negative for COVID-19 virus; cT value > 30. *The Cycle Threshold (cT) value is a marker of the viral load in a patient, potentially suggesting the severity of the COVID-19 infection. The lower the cT value, the higher the severity. Medical Staff is from the HMC and PHCC (included as part of LOC)^*^

**Table 2:** Summary of outdoor mass events hosted by Qatar other than football in 2020-2021

Tournaments	Duration	Event	Spectators

**Qatar Classic Squash Championship**	1-7, November 2020	International	Yes*

**Qatar International Handball Tournament**	25-29, December 2020	International	No

**Qatar Open Amateur Golf Championship**	25-27, February 2021	Local	No

**Commercial Bank CHI AL SHAQAB Equestrian Competition**	25-27, February 2021	International	Yes**

**Qatar Total Open (Tennis) Professional Women Tennis Tournament**	1-6, March 2021	International	Yes*

**Longines Global Champions Tour Equestrian Competition**	4-6, March 2021	International	Yes**

**Qatar Exxon Mobil Open Professional Men’s Tennis Tournament**	8-13, March 2021	International	Yes*

**Qatar Masters Golf Tournament**	11-14, March 2021	International	Yes*

**Qatar motorcycle Grand Prix**	26-28, March 2021	International	Yes**

**Tissot Grand Prix of Doha**	2-4, April 2021	International	No


*With 20% spectator attendance; ** with 10% spectator attendance
